# Fractalkine (CX3CL1) and Its Receptor CX3CR1 May Contribute to Increased Angiogenesis in Diabetic Placenta

**DOI:** 10.1155/2013/437576

**Published:** 2013-07-16

**Authors:** Dariusz Szukiewicz, Jan Kochanowski, Michal Pyzlak, Grzegorz Szewczyk, Aleksandra Stangret, Tarun Kumar Mittal

**Affiliations:** ^1^Department of General & Experimental Pathology, Second Faculty of Medicine, Medical University of Warsaw, Ulica Krakowskie Przedmiescie 26/28, 00-928 Warsaw, Poland; ^2^Department of Neurology, Second Faculty of Medicine, Medical University of Warsaw, Ulica Ceglowska 80, 01-809 Warsaw, Poland; ^3^Department of Obstetrics & Gynecology, Second Faculty of Medicine, Medical University of Warsaw, Ulica Kondratowicza 8, 03-242 Warsaw, Poland

## Abstract

Chemokine CX3CL1 is unique, possessing the ability to act as a dual agent: chemoattractant and adhesive compound. Acting via its sole receptor CX3CR1, CX3CL1 participates in many processes in human placental tissue, including inflammation and angiogenesis. Strongly upregulated by hypoxia and/or inflammation-induced inflammatory cytokines secretion, CX3CL1 may act locally as a key angiogenic factor. Both clinical observations and histopathological studies of the diabetic placenta have confirmed an increased incidence of hypoxia and inflammatory reactions with defective angiogenesis. In this study we examined comparatively (diabetes class C complicated versus normal pregnancy) the correlation between CX3CL1 content in placental tissue, the mean CX3CR1 expression, and density of the network of placental microvessels. A sandwich enzyme immunoassay was applied for CX3CL1 measurement in placental tissue homogenates, whereas quantitative immunohistochemical techniques were used for the assessment of CX3CR1 expression and the microvascular density. Significant differences have been observed for all analyzed parameters between the groups. The mean concentration of CX3CL1 in diabetes was increased and accompanied by augmented placental microvessel density as well as a higher expression of CX3CR1. In conclusion, we suggest involvement of CX3CL1/CX3CR1 signaling pathway in the pathomechanism of placental microvasculature remodeling in diabetes class C.

## 1. Introduction

Augmented immune tolerance during pregnancy prevents onset of inflammatory immune responses that may cause fetal rejection [1]. Specific roles of the cytokine network in the human placenta include local modulation of the balance between pro and anti-inflammatory factors [2]. Thus, together with pregnancy-specific hormones, adequate participation of placental cytokines in immune control is crucial for normal intrauterine growth of the fetus [3]. Since many mediators of inflammation are angiogenic, any disorder associated with the shift in the precise quantitative balance between proinflammatory cytokines and their inhibitors may influence the development of placental vessels [4]. Pathomechanisms of diabetes mellitus during pregnancy include changed oxygen and metabolic pathways, resulting in abnormal placental villous growth and function [5]. It was reported that dysregulation of angiogenic response within diabetic placental tissue may significantly affect fetal well-being by an increase in susceptibility to hypoxia and hypoxia-associated apoptotic triggers [6]. 

Clinical observations and histopathological studies of the placenta have confirmed an increased incidence of inflammatory reactions in diabetes [7, 8]. The class C of diabetes in pregnancy (after White) is the last stage without recognized vascular morphological changes in light microscopy [9] (Table 1). We previously showed that increased density of the villous vascular network in class C human diabetic placenta was correlated with higher histamine and vascular endothelial growth factor (VEGF) concentrations and increased number of placental mast cells [10, 11]. Since several mastocyte-derived mediators, including histamine, are angiogenic and regulate endothelial cell proliferation and function, degranulation of mast cells may augment local angiogenesis [12]. One explanation for the increase in mast cell number is migration of mast cells in diabetic placental tissue. Considering this, the role of chemotactic cytokines—chemokines in the pathogenesis of diabetes-induced neovascularization—should be suspected [13].

Chemokines form a superfamily of cytokines with the major roles involved in the modulation of immune response and the guidance of migrating leukocytes towards or away from the chemotactic substance (chemoattractant or chemorepellent activities, resp.) [14]. First description of chemokine CX3CL1 (known also as fractalkine or neurotactin) was given in 1997 by Bazan et al. and Pan et al. [15, 16]. To date, encoded on human chromosome 16 and possessing three amino-acid residues located between the first two cysteine residues in the molecule, CX3CL1 is the lone member of the CX3C (delta) subfamily of chemokines [17]. Unlike other chemokines, CX3CL1 is of nonhaemopoietic origin and exists in a soluble form as chemotactic protein and in a membrane-anchored form mainly on endothelial cells as a cell-adhesion molecule. The main roles of the soluble CX3CL1 domain include chemotactic activity for natural killer (NK) cells, T cells, monocytes, and mast cells, but not neutrophils, whereas membrane-anchored form of the chemokine is involved in promotion of leukocyte binding and adhesion. This dual function (chemoattractant and adhesive compound) makes CX3CL1 unique among the known chemokine subfamilies [18]. Together with some other chemokines (CCL4, CCL7, CCL14) CX3CL1 participates in the processes of implantation, trophoblast invasion into the spiral uterine arteries, placental angiogenesis, response to inflammatory and immunologic factors within the uteroplacental unit, and induction of labor [19–21]. Endothelial cells of the placental vasculature, vascular smooth muscle cells, and amniotic epithelial cells are the main sources of CX3CL1 in the human placenta including the membranes [22–24].

Actions of CX3CL1 are mediated by its sole receptor CX3CR1 (previously known as V28), G*α*i protein-linked seven-transmembrane receptor [25]. Expression of CX3CR1 was confirmed in endothelial cells, as well as on mast cells and other cell types, including monocytes, NK cells, microglial cells, neurons, and subpopulations of T-lymphocytes [26]. CX3CR1 receptor stimulation leads to the activation of both CX3CL1-dependent and integrin-dependent migrations of cells with augmented adhesion in result of synergistic reactions [27]. 

Strongly upregulated by hypoxia and/or inflammation-induced inflammatory cytokines secretion, especially tumor necrosis factor alpha (TNF*α*), interferon gamma (IFN*γ*), and interleukin-1 beta (IL-1*β*), CX3CL1 may act locally as a key angiogenic factor [28]. It was reported that activation of CX3CL1/CX3CR1 signaling pathway induces angiogenesis through two sequential steps: the induction of hypoxia inducible factor 1 alpha (HIF-1*α*) and vascular endothelial growth factor-(VEGF-) A gene expression and the subsequent VEGF-A/vascular endothelial growth factor receptor type 2 (VEGFR2 or KDR) induced angiogenesis [29, 30].

The aim of this study was to examine comparatively (diabetes class C—complicated versus normal pregnancy) the correlation between CX3CL1 content in placental tissue, the mean CX3CR1 expression, and density of the network of placental microvessels. 

## 2. Materials and Methods

The study was conducted in compliance with international and local laws of human experimentation and was officially approved by local ethics committee, and written consent from the women was obtained for use of their placentae. Strictly speaking, the work has been carried out in accordance with the code of ethics of the World Medical Association (Declaration of Helsinki) for experiments involving humans and the uniform requirements for manuscripts submitted to biomedical journals have been fulfilled.

### 2.1. Placental Tissue Collection

Eleven placentae obtained from nulliparas after single pregnancies complicated by diabetes class C (group I; the mean gestational age 250 ± 8 days), were compared with 11 placentae obtained from gestationally matched near-term controls (group II; 253 ± 4 days). All pregnancies were terminated by cesarean sections due to the fetal interest (group I), including breech presentation (pelvic longitudinal lie of the fetus) in group II. The control of glycemia in all cases was satisfactory; the levels of fraction of glycosylated hemoglobin in all trimesters of diabetic pregnancy were kept within the normal range (5%–7.5%). The courses of pregnancies in group II were normal, except for a near-term initiation of the contractile activity of the uterus. More detailed clinical characteristics of the two studied groups are given in Table 1.

**Figure 1:**
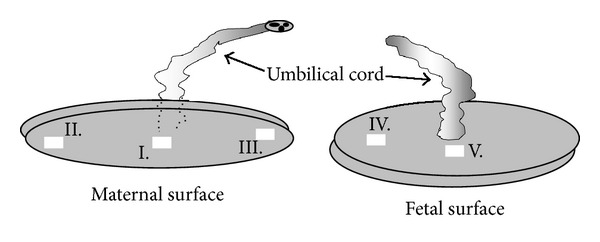
Location of samples collected in standardized manner from the maternal surface of the placenta (I–III) and from the fetal surface (IV-V). The mean weight of the sample: 10.49 ± 0.89 g.

Five specimens were excised in a standardized manner from each placenta: three from the region contiguous to the maternal surface (the first one—from the central part, the next two—from peripheral regions of the placental maternal surface) and two specimens from the region contiguous to the fetal surface (the first—from place of umbilical cord insertion, the next—from the peripheral region) (Figure 1). The tissue material obtained by this procedure was subjected either to freezing in carbon dioxide snow for CX3CL1 concentration measurement or fixed in paraffin wax and cut at 5 *μ*m, before staining procedures with hematoxylin/eosin and immunohistochemistry. 

### 2.2. Measurement of CX3CL1 Content

Concentration of CX3CL1 was estimated in the frozen placental excisions. During handling, the material was kept on ice. Before generating a lysate, the tissue was initially cut into about 1 mm^3^ cubes by using a razor blade on a glass plate held on ice. In order to perform a gentle cell disruption, the cubes were then transferred into a hand-held Potter S homogenizer. The liquefied tissue was placed in the 1.5 mL tubes and centrifuged at 13000 rpm for 3 min at 4°C. The clear supernatant was used for in vitro quantitative measurement of CX3CL1 in the placental tissue homogenates by a sandwich enzyme immunoassay. Chemokine C-X3-C-Motif Ligand 1 (CX3CL1) BioAssay ELISA Kit (Human; Cat. no. 024096) was applied with detection range 0.156–10 ng/mL and sensitivity of 0.053 ng/mL. The mean value was calculated for each examined placenta and expressed in pg/g of wet tissue weight.

### 2.3. Immunohistochemistry of CX3CR1

Human placenta paraffin 5 *μ*m sections were subjected to the standard immunohistochemical procedures that led to visualization of CX3CR1. Rabbit polyclonal antibody IgG to CX3CR1 (ab8020; Abcam Inc., USA; concentration of 10 *μ*g/mL) was used as primary and goat anti-rabbit IgG as biotinylated secondary antibody (ab64256; Abcam; 0.5% v/v). In order to visualize the primary anti-CX3CR1 antibodies, the StreptABComplex/HRP Duet (Dako Cytomation, Glostrup, Denmark) was used, following the protocol recommended by the manufacturer, with 3,3′-diaminobenzidine that served as a chromogen. The respective negative controls for immunostainings were prepared simultaneously by replacement of the polyclonal primary antibody by normal rabbit preimmune IgG diluted with phosphate buffered saline, containing 3% bovine serum albumin at the same protein concentration as that used for the primary antibody.

### 2.4. Mean Density of Placental Microvessels

Identification of the vasculature elements in placental sections was performed using endothelial cell marker, rabbit polyclonal antibody anti-CD31 (dilution 1 : 50, ab28364; Abcam Inc., Cambridge, MA, USA). The tissue was incubated with the primary antibody for 30 minutes. Next, a biotinylated goat anti-rabbit antibody was used as the secondary (Abcam). 

Using light microscopy with computed morphometry for quantitative analysis (Quantimet 500C+ image analysis workstation provided by Leica, UK), the vascular/extravascular tissular index (V/EVTI) was estimated in calibrated areas of the placental sections. Each preparation (paraffin section) underwent three area analyses repeated by two experienced, independent observers. The single area measured with the picture analyzer amounted to 721320 *μ*m^2^ and the total number of preparations 55 per group. The picture analysis procedure consisted in a measurement of the total vascular area. Consequently, the total lumen area of all types of identified vessels was summed up in both groups. With the purpose of a minimizing disruption caused by technical errors, especially unaxial section of the vessel, the lowest value of Ferret's diameter was accepted as the diameter of single lumen. Thus, V/EVTI represents the ratio, which reflects intensity of vascularization and is most closely correlated with the mean density of placental microvessels [31]. 

### 2.5. Expression of CX3CR1

After immunostaining, a quantitative immunohistochemistry based on morphometric software (Quantimet 500C+) was applied for CX3CR1 receptors identification in paraffin 5 *μ*m sections of the placental specimens under light microscopy. All morphometric procedures were carried out twice by two independent researchers and the average values uploaded in the result recording tab. Intensity of immunostaining was evaluated using mean colour saturation parameter and thresholding in grey-level histograms. Thus, expression of CX3CR1 corresponded to the total immunostained calibrated area of examined sections, where colour saturation comprises segmentation-separation criteria for objects. Single analysed image area amounted to 138692 *μ*m^2^ (magnification ×200). In total, 165 visual fields have been analysed (15 visual fields per placenta) in each studied group. To achieve maximum accuracy of measurements, the following factors have been controlled or monitored: averaging of image intake, hue, illumination, luminance, power supply, relation of illumination to quantification of area percentage of positively staining structures, shading correction, and warming up. More detailed description of these morphometric procedures was given previously elsewhere [31, 32]. Assuming that the accuracy of CX3CR1 expression measurement may be significantly affected by the local differences in density of placental microvessels, in both groups we examined comparatively vascular density-matched samples with the tolerated range of discrepancy ±5% [33]. Morphometric results comprising 90% confidence intervals were reported as mean percentage values ± SEM.

### 2.6. Statistical Analysis

Statistical analyses were performed using Statistica 8.0 software (Stat-Soft, Poland). Mann-Whitney's *U* test was applied. The results were expressed as means ± SEM or mean percentage values ± SEM. Differences between group I (diabetes class C) and group II (normal-course pregnancy) were deemed statistically significant if *P* < 0.05. 

## 3. Results

**Figure 2:**
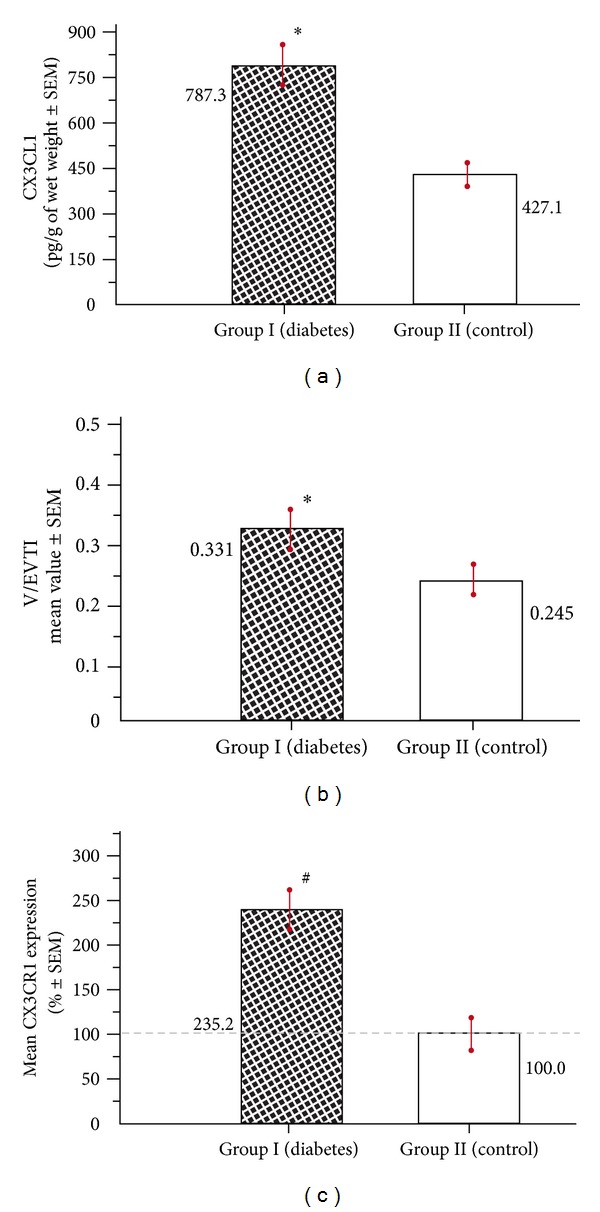
Placental samples collected after diabetes class C (group I) versus specimens obtained after normal pregnancy (group II): (a) the mean concentration of CX3CL1; (b) density of the microvessels estimated using the mean vascular/extravascular tissular index (V/EVTI); (c) the mean percentage expression of CX3CR1. The values obtained for normal pregnancy (0.016 ± 0.0014; abstract numbers ± SEM) were taken as 100% (*P < 0.05; ^#^ *P* < 0.01).

The results pertaining to CX3CL1 content in placental tissue, the mean density of microvessels, and CX3CR1 expression are summarized in Figures 2(a), 2(b), and 2(c), respectively. Significant differences have been observed for all analyzed parameters between the groups. 

The mean concentration of CX3CL1 in placental cuts from the diabetic group (group I) was increased compared to controls (group II) and amounted to 787.3 ± 70.2 pg/g of wet weight ± SEM versus 427.1 ± 37.4 (Figure 2(a)). There were no significant differences in CX3CL1 levels between the specimens collected from maternal and fetal surface of the placenta within the group. 

The mean V/EVTI value (absolute number ± SEM) in placental tissue obtained after diabetes class C-complicated pregnancies was increased (*P* < 0.05), amounting to 0.331 ± 0.037 versus 0.245 ± 0.025 in normal controls (Figure 2(b)). These mean values of V/EVTI were evaluated totally for each group, using the partial data gained from all collected placental samples. However, we observed in both groups that in the samples obtained from the maternal surface, the mean density of microvessels expressed as V/EVTI was significantly higher (*P* < 0.05) compared to the mean V/EVTI calculated for the specimens of placental tissue obtained from the fetal surface (the data not shown on the chart).

**Figure 3:**
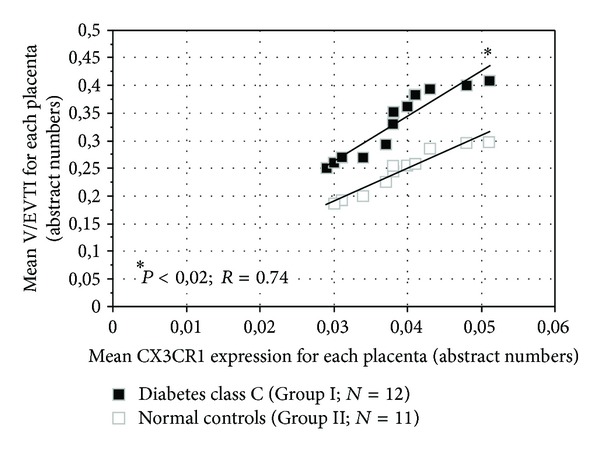
The mean values of V/EVTI versus the mean CX3CR1 expressions: diabetic and normal placentae, comparatively.

Evaluation of the relationship between the mean CX3CR1 expression and the mean V/EVTI revealed strong positive correlation and significant differences between the groups (Figure 3). The higher expression of CX3CR1 in diabetes corresponded to the augmentation of the placental vascularization, as assessed by V/EVTI.

**Figure 4:**
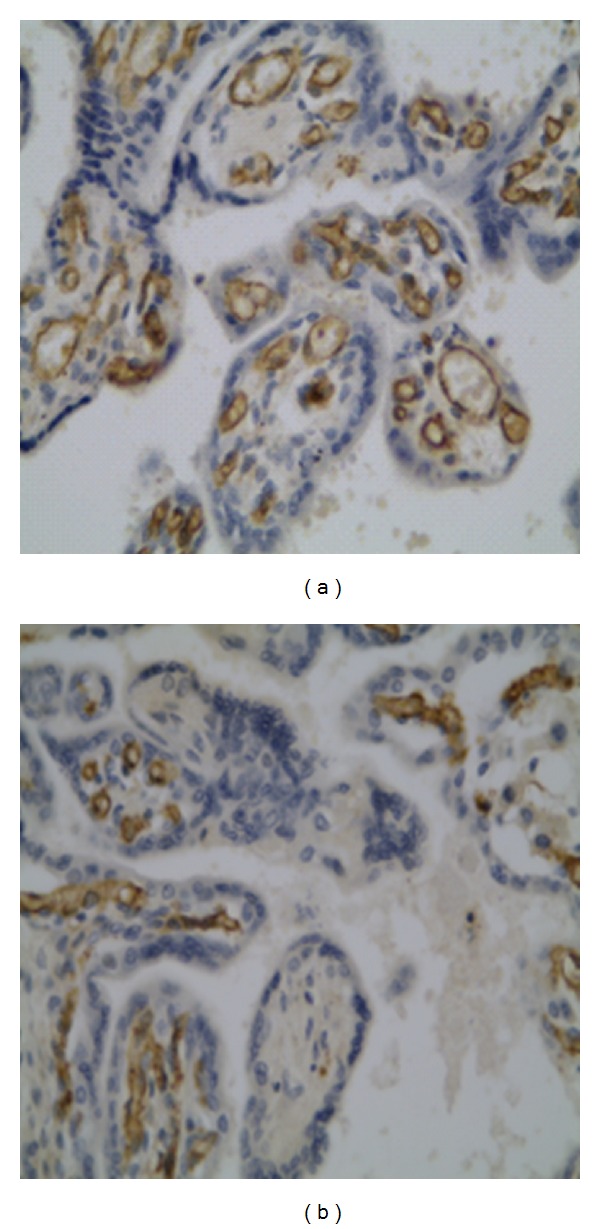
Immunohistochemical localization of the receptor CX3CR1 in the placental tissue at 400x magnification: (a) diabetes class C (group I); (b) normal controls (group II).

Immunohistochemical technique used for identification of CX3CR1 revealed that this receptor is predominantly located in placental endothelial cells (Figure 4). The mean percentage value of CX3CR1 expression estimated in the vascular density-matched samples was remarkably higher (*P* < 0.01) in diabetes and reached 235.2 ± 24.4 (%, ±SEM) of the reference value established in group II (Figure 2(c)).

## 4. Discussion

The growth, maintenance and functioning of placental microvessels may be affected by the majority of the pathological changes in maternal/fetal hemodynamics and by alterations in blood composition or content [34]. Different authors working independently have reported that the induction of the pro-inflammatory milieu is inherently involved in the pathophysiology of diabetes mellitus, including diabetic placenta [7, 35]. In such an environment, frequent episodes of local hypoxia and transient hyperglycemia (even in well-controlled cases) are observed and are associated with elevated levels of free oxygen radicals, advanced glycation end products (AGEs), and some proinflammatory cytokines, especially those with angiogenic properties [6, 35]. However, it has also been reported that placental expression of the inflammatory cytokines in response to oxidative stress is significantly reduced in gestational diabetes mellitus [36]. This study did not involve women with diabetes class C, and CX3CL1 concentrations were not examined.

Secondary to hypoxia, increases in the local levels of TNF*α*, IFN*γ*, and IL-1*β* potentiate the angiogenic action of CX3CL1 that is simply correlated with an increase in the concentration of this chemokine [28, 29, 37]. CX3CL1 stimulates ex vivo and in vivo angiogenesis in a dose- and time-dependent manner and acts on endothelial cells even more strongly than the well-known angiogenic factor VEGF [29]. It has been reported that CX3CL1 (100 nM) induced endothelial cells to form capillary tubes in synthetic matrix with at least the same efficiency as the angiogenic mediators bFGF (60 nM) and VEGF (100 nM) [38]. Based on the data that are available to date, there is no clear standpoint on the roles of VEGF and its receptor VEGFR-2 (KDR/Flt-1) in CX3CL1-mediated angiogenesis. Some authors have reported that the activation of CX3CL1/CX3CR1 by vascular endothelial cells induces angiogenesis through VEGF-A/KDR. Increased VEGF production can be achieved through the pathways involving hypoxia-inducible factor-1 alpha (HIF-1*α*) and p42/44 mitogen-activated protein (MAP) kinase [29]. In our previous study of the diabetic placenta, we found that higher VEGF expression levels were positively correlated with increased microvascular density [11]. However, the results of another study indicate that the mechanism of CX3CL1 expression and its angiogenic effects differ from those of VEGF. Whereas hypoxia strongly induces VEGF expression, an ischemic environment actually inhibits the expression of CX3CL1. This finding may suggest that hypoxia promotes VEGF-mediated angiogenesis, while inflammation involves CX3CL1-related new vessel formation [39]. Considering this, our findings are rather correlative and not mechanistic in nature. The results provide no formal evidence for the causal involvement of the CX3CL1/CX3CR1 system in altered placental vascularity, especially because we have previously reported changes in the placental VEGF expression [11].

Studies of the molecular mechanism by which CX3CL1 regulates angiogenesis in human umbilical vein endothelial cells (HUVEC) showed that CX3CL1 did not increase the levels of VEGF mRNA. These results indicate that CX3CL1 may act as a direct angiogenic modulator in endothelial cells without inducing VEGF expression. It is likely that the angiogenic activities of CX3CL1 and VEGF are independent of one another but utilize the Raf1/MEK/ERK kinase cascade and P13 K/Akt/eNOS activation as common mediators in their angiogenic signaling pathways. In other words, it was proposed recently that CX3CL1, acting directly on angiogenesis via CX3CR1, may activate the two previously mentioned distinct signaling pathways [40].

All of the characteristic steps in the complex process of new vessel formation are augmented after CX3CL1 treatment, including endothelial cell proliferation, migration, and tube-like structure formation [30, 41]. 

The statistically significant upregulation of CX3CR1 expression documented in diabetic endothelial cells should also be considered, particularly because of the existence of an autoregulatory mechanism between CX3CL1 and CX3CR1 [42]. A form of autoregulation between CX3CR1 and CX3CL1 via the autocrine loop (CX3CR1/CX3CL1 axis) was proposed by independent authors with respect to many cell types, including endothelial cells [43–45]. So far, however, there is no convincing scientific evidence that the same human endothelial cells possess the ability to produce CX3CL1 and simultaneously express CX3CR1. However, because CX3CR1 is the only known receptor for CX3CL1, it is possible that CX3CL1-induced endothelial cell migration and capillary tube formation was mediated through the interaction between CX3CL1 and its endothelial receptor CX3CR1 in an autocrine manner [38]. 

It is worth noting that the CX3CR1 signal documented in Figure 4 and considered to be associated with endothelial cells may also come from pericytes. A double-staining technique or high-resolution, high-power imaging should be implemented in future studies to elucidate this issue. 

An excess of CX3CL1 and other inflammatory mediators may be responsible for augmented angiogenesis within the placental unit. According to the results of other studies, the regulation of CX3CL1 function may be maintained on both protein and gene expression levels. Increased expression of matrix metalloproteinases (MMPs) and disintegrin metalloproteinases (ADAMs, including ADAM17/TACE) or sheddases have been reported in the diabetic placenta [46, 47]. These increases may lead to an increase in the concentration of the soluble form of CX3CL1, which is derived from its membrane form. 

In contrast, the proinflammatory background of diabetes, which includes increased placental levels of TNF*α*, IFN*γ*, and IL-1*β*, promotes enhanced CX3CL1 gene expression [48]. 

Further studies are needed to determine which mechanism of CX3CL1 regulation predominates in diabetes class C.

The classic example of a diabetic complication with increased defective angiogenesis mediated by CX3CL1 with the inflammatory background is proliferative retinopathy [38, 49].

Both in this study and in previous reports, we have provided evidence of altered vascularization in the diabetic placenta. The results of the present investigation strongly support the hypothesis that CX3CL1-related pathways is involved in the pathomechanism of placental angiogenesis in diabetes. Increased V/EVTI values are consistent with our previous approach to evaluating the density of placental microvessels in diabetes class C with respect to histamine concentration and mast cell number [10, 11]. 

Chemotaxis assays performed in vitro by other researchers have demonstrated that increased CX3CL1 levels initiate and augment the migration of specific CX3CR1-positive subpopulations of inflammatory cells [50]. For example, we previously observed both an increase in the number of mast cells in the diabetic placenta and changes in their heterogeneity, namely, a shift of the quantitative balance between tryptase-positive and tryptase/chymase-positive cells [51]. This shift may influence the placental cytokine network profile in diabetes, possibly including an increase in the local CX3CL1 concentration. Considering the dual role of CX3CL1, mechanisms that influence placental angiogenesis and are related to the direct action of CX3CL1 and the chemoattractant properties of CX3CL1 should be considered. Interestingly, despite the significant expression of CX3CR1 by human mast cells, CX3CL1 does not directly produce mast cell degranulation [52]. Thus, CX3CL1 should not be simply linked with mast cell-mediated angiogenesis [12].

It should be clearly stated that altered vascular patterns are not necessarily a result of angiogenesis and could represent a form of vascular remodeling rather than overt capillary growth. This phenomenon is illustrated by the larger, rather than more numerous vessels depicted in Figure 4. The algorithm used to enumerate vascular changes does not permit the capturing of microvessels [53].

In conclusion, it is very likely that increased CX3CL1 concentration in diabetes class C, together with the upregulation of its specific and sole receptor, CX3CR1, are involved in the pathomechanism of placental microvasculature remodeling. Further studies are needed to elucidate the rationale for anti-CX3CL1 or anti-CX3CR1 therapies during diabetic pregnancy. 

## Figures and Tables

**Table 1:** Clinical characteristics of the two studied groups: diabetes class C after White (group I) versus normal-course pregnancies (group II).

Parameter	Group I (diabetes class C)	Group II (normal controls)
Number of patients/placentae/newborns (*n*)	11/11/11	11/11/11
Age of the patients in full years (range; mean; median)	26–33; 29; 28	22–33; 27; 26
Parity	0	0
Gestational age in days (range; mean; median)	242–258; 250; 252	249–257; 253; 255
Method of delivery	Cesarean section	Cesarean section
1st minute Apgar's score (range; mean)	8–10; 9.3	9-10; 9.7
Blood pressure during pregnancy	All records within normal range^a^	All records within normal range^a^
Proteinuria during pregnancy	Not present^b^	Not present
Liver blood tests (aminotransferases enzymes AST and ALT levels)	Within normal range^c^	Within normal range^c^
Smoking during pregnancy	1 declared >5 cigarettes per day	2 declared >5 cigarettes per day
Body Mass Index <21 or >35	None	None
Other identified risk factors	None	None
Birth weight in grams (range; mean; median)	2995–3590; 3386; 3277	3056–3440; 3307; 3320
Newborns gender (M—male; F—female)	4M + 7F	6M + 5F
Weight of placenta in grams (range; mean, median)	425–616; 571; 563	482–626; 554; 539

^a^The normal range of the blood pressure was defined as systolic pressure between 100 and 140 mm Hg and diastolic pressure between 60 and 90 mm Hg.

^b^Microalbuminuria (urinary albumin excretion in the range of 30–300 mg/24 h) not present.

^c^The normal range of values for AST is from 5 to 40 units per liter of serum, and the normal range of values for ALT is from 7 to 56 units per liter of serum.
